# Mental Health of University Students during the COVID-19 Pandemic – A Prospective Cross-National Study

**DOI:** 10.1192/j.eurpsy.2023.67

**Published:** 2023-07-19

**Authors:** D. Ochnik

**Affiliations:** Department of Clinical Psychology, Faculty of Medicine, Academy of Silesia, Katowice, Poland

## Abstract

**Abstract:**

This prospective study aimed to examine changes in mental health and differences due to educational status (ES) and country among young adults aged 20-40 from four countries during the COVID-19 pandemic in a three-month period.

The total of 1714 participants (932 women): students (*n* = 321) and non-students (*n* = 519) aged 20-30, educated (*n* = 388), and non-educated (*n* = 486) adults aged 31-40 from Poland (*n* = 445), Slovenia (*n* = 430), Germany (*n* = 417), and Israel (*n* = 422) responded to online survey in February 2021 and May–June 2021. The used measurements were: Perceived Stress Scale (PSS-10), Generalized Anxiety Disorder (GAD-7), and Patient Health Questionnaire (PHQ-8).

A repeated-measures two-way mixed-factor ANOVA was performed to examine changes over time, educational status (ES), and across countries for mental health indicators. The results showed stability over time in anxiety and depression while a small decrease in stress. Students scored significantly higher in stress, anxiety, and depression compared to non-/educated adults and in depression compared to non-studying peers. Participants from Poland and Germany scored higher in anxiety and depression than from Slovenia and Israel. Moreover, Polish participants reported the highest stress among all countries.

The student population is more vulnerable to mental health issues than non-studying peers and adults with and without an academic degree, particularly in Poland and Germany.

**Disclosure of Interest:**

None Declared

